# Health‐Seeking Behaviours Among Adolescent Girls and Young Women With Sexually Transmitted Infections: Evidence From Sub‐Saharan Africa

**DOI:** 10.1002/puh2.70088

**Published:** 2025-08-18

**Authors:** Bright Opoku Ahinkorah, Richard Gyan Aboagye, Irene Esi Donkoh, Joshua Okyere, Sanni Yaya

**Affiliations:** ^1^ Faculty of Health and Medical Sciences The University of Adelaide Adelaide Australia; ^2^ Department of Family and Community Health Fred N. Binka School of Public Health University of Health and Allied Sciences Hohoe Ghana; ^3^ Department of Medical Laboratory Science University of Cape Coast Cape Coast Ghana; ^4^ Department of Population and Health University of Cape Coast Cape Coast Ghana; ^5^ Department of Allied Health Professions, Sport and Exercise School of Human and Health Sciences University of Huddersfield Huddersfield West Yorkshire UK; ^6^ The George Institute For Global Health, Imperial College London London UK

**Keywords:** adolescent girls, Demographic and Health Survey, healthcare seeking, sexually transmitted infections, sub‐Saharan Africa, young women

## Abstract

**Background and Aims:**

In sub‐Saharan Africa (SSA), less than 10% of men, compared to 50%–80% of women, are affected by sexually transmitted infections (STIs). Untreated STIs can lead to severe reproductive health complications, including infertility, pelvic inflammatory disease, and increased susceptibility to HIV. Understanding the determinants of healthcare‐seeking behaviour for STIs is crucial for informing policies and interventions aimed at improving access to timely and appropriate care. This study examined the healthcare‐seeking behaviours of adolescent girls and young women (AGYW) with STIs in SSA.

**Methods:**

Our study utilised data from the Demographic and Health Surveys of 20 countries in SSA. We used a forest plot to present the results on the prevalence of healthcare seeking for STIs among AGYW in SSA. Additionally, we examined the predictors of healthcare seeking for STIs using a multilevel binary logistic regression analysis.

**Results:**

The results showed that 54.31% (95% confidence interval [CI]: 53.48–55.14) of AGYW in SSA sought healthcare for STIs. This ranged from as low as 26.98% (95% CI: 23.44–30.52) in Ethiopia to as high as 82.50% (95% CI: 78.38–86.62) in Liberia. AGYW aged 20–24 (adjusted odds ratio [aOR] = 1.49, 95% CI: 1.31–1.71), those who were cohabiting (aOR = 1.40, 95% CI: 1.10–1.79), those with secondary [aOR = 1.49, 95% CI: 1.20–1.85] or higher education [aOR = 1.68, 95% CI: 1.08–2.61], those who were working at the time of the survey [aOR = 1.23, 95% CI: 1.07–1.40], those who were covered by health insurance [aOR = 1.45, 95% CI: 1.09–1.93], and those in richest wealth quintiles [aOR = 2.18, 95% CI: 1.62–2.92] were more likely to seek healthcare for STIs.

**Conclusion:**

Our study has shown that the proportion of AGYW who sought healthcare for their STIs is relatively low, with country‐level variations. Several factors were found to be associated with healthcare seeking for STIs. Focused interventions are required to enhance access to healthcare treatments for STIs among the vulnerable sub‐populations.

## Graphical abstract

Only 54% of adolescent girls and young women in sub‐Saharan Africa seek care for sexually transmitted infections. Education, wealth, health insurance, age, marital status, employment status, and radio exposure increase the likelihood of seeking care, whereas rural residence and regional disparities hinder access. These resultshighlight the urgent need to esnure equity and address the communication gaps in healthcare‐seeking behaviour for sexually transmitted infections.

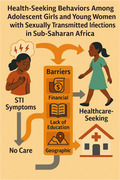

## 1 Introduction

Sexually transmitted infections (STIs) are considered a significant public health concern, with over a million new infections acquired daily across the globe [1]. Statistical reports from the World Health Organization (WHO) further indicate nearly 374 million new STI cases per year [1]. The situation is endemic in sub‐Saharan Africa (SSA), where most cases originate [2, 3]. Consequently, eliminating STIs has become an important  component of the global public health priorities. This is evident in the WHO's renewed resolve to curb STIs in achieving global sexual and reproductive health [4], as well as in the Sustainable Development Goal (SDG) 3 [5, 6].

Despite the recent global dedication to end STIs, it continues to be highly prevalent among adolescent girls and young women (AGYW) [5]. The WHO indicates that there has not been a reduction in the new cases of human immunodeficiency virus (HIV) among AGYW between 2010 and 2015, and that this could pose a threat to ending the HIV pandemic [7]. Also, this could lead to increased morbidity and mortality in the future. Several factors have been found to contribute to AGYW's vulnerability to contracting STIs. These factors include multiple sexual partners, gender inequalities, poverty, early sexual debut, and poor access to sexual and reproductive health services [8, 9, 10].

Effective management of STIs among AGYW requires early detection, prompt treatment, and consistent preventive measures [5, 11]. However, the success of these interventions largely depends on the willingness of AGYW to seek healthcare services. Health‐seeking behaviours (HSB) refer to ‘any initiative taken by persons who perceive themselves to have a health issue for the purpose of finding an appropriate remedy’ [12]. To effectively design interventions to reduce STIs, it is essential to have a solid understanding of the HSB of AGYW who are infected with STIs.

Despite the importance of HSB, there is limited research on this topic among AGYW with STIs in SSA. The existing studies have been conducted in individual countries [13, 14, 15, 16, 17, 18]. The only study conducted at the regional level also focused on the self‐reported STIs among AGYW, but not their HSB [19]. As such, there is a lack of understanding of the situation at the regional level. This knowledge gap necessitates research to understand the regional dynamics with respect to the HSB of AGYW with STIs. This paper seeks to fill this gap by examining the healthcare‐seeking behaviours of AGYW with STIs in SSA. Findings from the study would provide evidence to help address STIs and support the achievement of SDG 3.

## 2 Methods

### 2.1 Data Source and Study Design

We wrote this article with reference to the Strengthening the Reporting of Observational Studies in Epidemiology (STROBE) guideline [20]. We used data from the recent Demographic and Health Survey (DHS) of 20 countries in SSA. Data for the study were extracted from the women's files in each of the 20 countries. As stipulated in the literature [21], the DHS is a nationwide survey conducted in over 90 low‐ and middle‐income countries globally. A two‐stage cluster sampling method was used to select survey respondents, with the detailed sampling methodology outlined in the literature [22]. Briefly, the DHS adopted a cross‐sectional design. In all participating countries, each region was divided into rural and urban areas. Next, clusters or enumeration areas (first stage) were selected using probability proportional to size within each rural and urban stratum. At the second stage, a household listing and mapping were conducted to aid in creating an updated list of households in each selected cluster. An estimated 28–30 clusters were randomly selected per cluster in each rural–urban stratum. The women in the selected households were included in the study. Only the women aged 15‐49 who were residents in the households (permanent residents) and those who were visitors but had spent the night before the survey and provided consent were included in the study. The DHS used a structured questionnaire to gather data from the respondents about health indicators, such as STIs and their HSB [21, 23]. Pretested structured questionnaires were used to collect data from the respondents. We analysed a weighted sample of 12,787 AGYW (15‐24 years) who had STIs before the survey.

### 2.2 Variables

#### 2.2.1 Outcome Variable

The outcome variable was healthcare seeking for STIs. In the DHS, respondents who had STIs were asked to indicate whether they sought care or treatment for their infection using the question: *When you had the infection, did you seek any kind of advice or treatment?* The infections in this context refer to any STIs and their symptoms, such as genital discharge, genital sores, and genital ulcers. Response options to this question were ‘0 = no’ and ‘1 = yes’ [13, 16, 17, 18].

#### 2.2.2 Exposure Variables

Fifteen exposure variables were considered in this study. The variables were selected based their association with healthcare seeking for STIs from previous studies [13, 16, 17, 18]. Moreover, only the variables found in the DHS dataset were used. We further grouped the variables into individual and contextual levels. The individual‐level variables consisted of the age of the respondent, educational level, marital status, current working status, exposure to watching television, exposure to listening to the radio, exposure to reading newspapers/magazines, health insurance coverage, getting medical help for self: getting money needed for treatment, getting medical help for self: permission to go, getting medical help for self: not wanting to go alone, and getting medical help for self: distance to health facility. Household wealth index, place of residence,‐ and geographical sub‐region were the contextual‐level variables. The categories of the exposure variables can be found in Table 1.

### 2.3 Statistical Analyses

**FIGURE 1:**
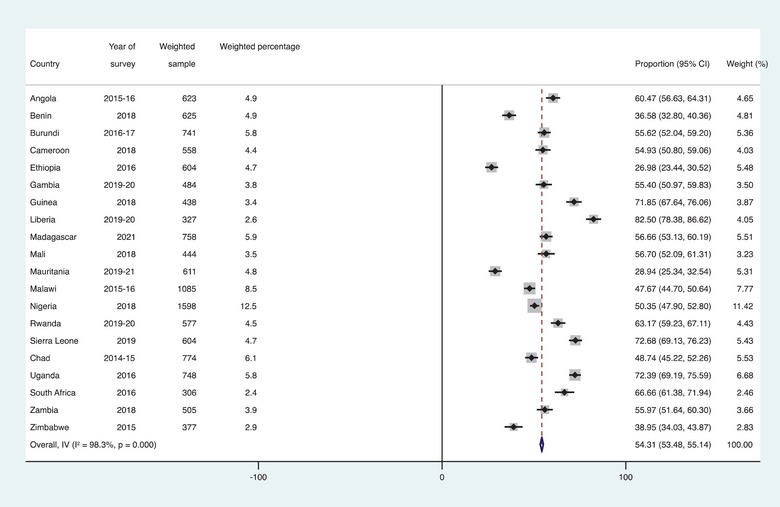
Prevalence of HSB among AGYW with STIs in SSA.

Data for the study were extracted and appended for final analysis using Stata version 17.0 (Stata Corporation, College Station, TX, USA). We cleaned the data in the individual country files, where all missing observations were dropped and weighted before appending. Later, we used a forest plot to present the results on the prevalence of healthcare seeking for STIs among AGYW in SSA (Figure 1). This was followed by a cross‐tabulation analysis to determine the distribution of healthcare seeking for STIs across the exposure variables included in the study. Further, we selected the exposure variables for inclusion in a multilevel binary logistic regression analysis using a binary logistic regression. The results of the binary logistic regression analysis were presented using crude odds ratio (cOR) with their 95% confidence intervals (95% CI). All variables with a *p*‐value less than 0.05 were considered statistically significant and included in the multilevel analysis. We performed a multilevel analysis using four models (Models I–IV) to examine the factors associated with HSB for STIs. Model I had no explanatory variables and was considered an empty model. We included the individual‐ and contextual‐level variables in Models II and III, respectively. Model IV was the complete model, which contained all the explanatory variables. We presented the multilevel regression analyses using adjusted odds ratio (aOR) with their 95% CIs. Additionally, we checked for model fitness and comparison using the Akaike information criterion (AIC), where the model with the least AIC value was considered the best‐fitted model. Hence, the results from the last model (Model IV) were presented in Section 3 and later discussed because it was the model with the least AIC value. The results of the multilevel analysis had two sections: fixed and random effects. The fixed effect results showed the association between the exposure variables and the outcome variable. The random effect results indicate the fitness of the models. Its results were presented using the AIC values and intra‐cluster correlation coefficient. All the analyses were weighted. The level of significance was set at *p*‐value less than 0.05.

### 2.4 Ethical Considerations

Ethical clearance was not sought for this study because the data used are freely available in the public domain. We obtained permission to use the DHS dataset for publication from the Monitoring and Evaluation to Assess and Use Results Demographic and Health Survey (MEASURE DHS). All detailed information concerning the DHS ethical issues can be accessed via http://goo.gl/ny8T6X.

## 3 Results

### 3.1 Prevalence of HSB Among AGYW in SSA

Figure 1 shows results on the proportion of AGYW who sought advice or treatment for STIs. The results showed that 54.31% (95% CI: 53.48–55.14) of AGYW in SSA sought healthcare for STIs. This ranged from as low as 26.98% (95% CI: 23.44–30.52) in Ethiopia to as high as 82.50% (95% CI: 78.38–86.62) in Liberia.

### 3.2 Distribution of HSB Among AGYW With STI in SSA

Table 1 presents the distribution of HSB among AGYW with STIs in SSA. Young women (20–24 years) had a significantly higher proportion (57.4%) of seeking advice/treatment for STIs compared to adolescents (15–19 years). For marital status, cohabiting AGYW had the highest proportion of seeking advice/treatment for STIs (59.8%); however, the difference was not statistically significant. Married AGYW, on the other hand, reported a significantly lower proportion of seeking advice/treatment (49.8%) compared to never‐married AGYW (56.0%). Regarding the level of education, the highest proportion of seeking advice/treatment for STIs was reported among AGYW with higher education (68.8%). Employed AGYW had a high proportion of seeking advice/treatment for STIs (55.2%) than those who were unemployed (52.2%).

**TABLE 1:** Distribution of HSB among AGYW with STI in SSA.

			Sought advice/treatment for STIs	
Variable	Weighted *N* = 12,787	Weighted (%)	**Yes (%)**	**cOR [95% CI]**
**Women's age (years)**				
15–19	4331	33.9	46.8	1.00
20–24	8456	66.1	57.4	1.53^***^ [1.36, 1.73]
**Marital status**				
Never married	4346	34.0	56.0	1.00
Married	5833	45.6	49.8	0.78^***^ [0.69, 0.88]
Cohabiting	1698	13.3	59.8	1.17 [0.96, 1.42]
Previously married	910	7.1	57.4	1.06 [0.81, 1.38]
**Educational level**				
No education	2551	20.0	43.3	1.00
Primary	4169	32.6	49.9	1.30^**^ [1.10, 1.54]
Secondary	5540	43.3	60.1	1.97^***^ [1.69, 2.29]
Higher	527	4.1	68.8	2.88^***^ [1.94, 4.28]
**Current working status**				
Not working	6102	47.7	52.2	1.00
Working	6685	52.3	55.2	1.12^*^ [1.01, 1.26]
**Exposed to watching television**				
No	7043	55.1	48.1	1.00
Yes	5744	44.9	60.7	1.66^***^ [1.48, 1.87]
**Exposed to listening to radio**				
No	5565	43.5	48.3	1.00
Yes	7222	56.5	58.0	1.48^***^ [1.31, 1.67]
**Exposed to reading newspaper or magazine**				
No	10,222	79.9	51.6	1.00
Yes	2565	20.1	62.4	1.55^***^ [1.33, 1.82]
**Getting medical help for self: getting permission to go**				
Big problem	2566	20.1	44.2	1.00
Not a big problem	10,221	79.9	56.2	1.61^***^ [1.39, 1.88]
**Getting medical help for self: getting money needed for treatment**				
Big problem	6679	52.2	50.3	1.00
Not a big problem	6108	47.8	57.5	1.34^***^ [1.19, 1.50]
**Getting medical help for self: distance to health facility**				
Big problem	4807	37.6	48.0	1.00
Not a big problem	7980	62.4	57.2	1.45^***^ [1.28, 1.64]
**Getting medical help for self: not wanting to go alone**				
Big problem	3366	26.3	45.7	1.00
Not a big problem	9421	73.7	56.6	1.55^***^ [1.34, 1.79]
**Covered by health insurance**				
No	11,901	93.1	52.7	1.00
Yes	886	6.9	67.6	1.87^***^ [1.49, 2.34]
**Wealth index**				
Poorest	2108	16.5	40.6	1.00
Poorer	2358	18.4	47.8	1.34^**^ [1.10, 1.64]
Middle	2557	20.0	47.6	1.33^**^ [1.09, 1.62]
Richer	2743	21.5	61.4	2.33^***^ [1.92, 2.83]
Richest	3021	23.6	65.9	2.83^***^ [2.32, 3.47]
**Place of residence**				
Urban	5032	39.35	63.1	1.00
Rural	7755	60.65	47.7	0.53^***^ [0.47, 0.60]
**Geographical sub‐regions**				
Southern Africa	306	2.4	66.7	1.00
Central Africa	1956	15.3	54.2	0.59^*^ [0.36, 0.96]
Eastern Africa	5395	42.2	53.0	0.56^*^ [0.36, 0.89]
Western Africa	5130	40.1	53.7	0.58^*^ [0.37, 0.91]

*Note:* 1.00 = reference category.

Abbreviations: CI, confidence interval; cOR, crude odds ratio.

* *p* < 0.05.

** *p* < 0.01.

*** *p* < 0.001.

Additionally, exposure to media had a positive association with seeking advice/treatment for STIs, with the highest proportions being reported among AGYW who read newspapers/magazines (62.4%), those who watched television (60.7%), and those who listened to the radio (58.0%). The highest proportion of AGYW who sought advice/treatment for STIs were those with health insurance coverage (67.6%), those in the richest wealth index (65.9%), as well as those who did not have a problem with getting permission to go to the health facility (56.2%), getting the needed money for treatment (57.5%), not having problem with the distance to the health facility (57.2%), and not having a problem with not wanting to go alone (56.6%).

Place of residence was also significantly associated with seeking advice/treatment for STIs, with AGYW living in rural areas (47.7%) having a significantly lower proportion of seeking advice/treatment compared to those living in urban areas (63.1%). Geographical sub‐regions showed varying proportions of seeking advice/treatment for STIs, with the lowest proportion being reported in Eastern Africa (53.0%) compared to those in Southern Africa (66.7%).

### 3.3 Factors Associated With HSB Among AGYW With STIs in SSA

Table 2 presents the results of the factors associated with HSB among AGYW with STIs in SSA. Our findings indicate that young women aged 20–24 [aOR = 1.49, 95% CI: 1.31–1.71], those who are cohabiting [aOR = 1.40, 95% CI: 1.10–1.79], those with primary [aOR = 1.25; 95% CI: 1.02–1.54], secondary [aOR = 1.49, 95% CI: 1.20–1.85] or higher education [aOR = 1.68, 95% CI: 1.08–2.61], those who are currently working [aOR = 1.23, 95% CI: 1.07–1.40], those who are covered by health insurance [aOR = 1.45, 95% CI: 1.09–1.93] and those in the richest wealth quintiles [aOR = 2.18, 95% CI: 1.62–2.92] were more likely to seek healthcare for STIs compared to their counterparts who were aged 15‐19, those who had never been in union, those with no education, those not working, those without health insurance, and those in the poorest wealth quintile households, respectively.

**TABLE 2:** Factors associated with HSB among AGYW STI in SSA.

Variable	Model I	Model II aOR [95% CI]	Model III aOR [95% CI]	Model IV aOR [95% CI]
**Fixed effect model**				
**Women's age (years)**				
15–19		1.00		1.00
20–24		1.53^***^ [1.34, 1.76]		1.49^***^ [1.31, 1.71]
**Marital status**				
Never married		1.00		1.00
Married		0.97 [0.82, 1.15]		1.07 [0.90, 1.27]
Cohabiting		1.28^*^ [1.01, 1.62]		1.40^**^ [1.10, 1.79]
Previously married		1.18 [0.88, 1.59]		1.29 [0.95, 1.75]
**Educational level**				
No education		1.00		1.00
Primary		1.19 [0.99, 1.43]		1.25^*^ [1.02, 1.54]
Secondary		1.68^***^ [1.37, 2.06]		1.49^***^ [1.20, 1.85]
Higher		2.16^***^ [1.39, 3.35]		1.68^*^ [1.08, 2.61]
**Current working status**				
Not working		1.00		1.00
Working		1.14 [1.00, 1.30]		1.23^**^ [1.07, 1.40]
**Exposed to watching television**				
No		1.00		1.00
Yes		1.33^***^ [1.15, 1.54]		1.01 [0.86, 1.19]
**Exposed to listening to radio**				
No		1.00		1.00
Yes		1.21^**^ [1.06, 1.39]		1.20^**^ [1.05, 1.38]
**Exposed to reading newspaper or magazine**				
No		1.00		1.00
Yes		1.10 [0.92, 1.33]		1.07 [0.89, 1.29]
**Getting medical help for self: getting money needed for treatment**				
Big problem		1.00		1.00
Not a big problem		1.05 [0.90, 1.23]		1.03 [0.88, 1.20]
**Getting medical help for self: distance to health facility**				
Big problem		1.00		1.00
Not a big problem		0.99 [0.83, 1.19]		0.92 [0.77, 1.11]
**Getting medical help for self: getting permission to go**				
Big problem		1.00		1.00
Not a big problem		1.21 [0.99, 1.49]		1.25^*^ [1.02, 1.54]
**Getting medical help for self: not wanting to go alone**				
Big problem		1.00		1.00
Not a big problem		1.28^*^ [1.06, 1.54]		1.25^*^ [1.03, 1.50]
**Covered by health insurance**				
No		1.00		1.00
Yes		1.42^*^ [1.07, 1.89]		1.45^*^ [1.09, 1.93]
**Wealth index**				
Poorest			1.00	1.00
Poorer			1.48^***^ [1.19, 1.85]	1.40^**^ [1.12, 1.77]
Middle			1.30^*^ [1.03, 1.64]	1.15 [0.91, 1.47]
Richer			2.14^***^ [1.68, 2.73]	1.81^***^ [1.40, 2.35]
Richest			2.69^***^ [2.06, 3.52]	2.18^***^ [1.62, 2.92]
**Place of residence**				
Urban			1.00	1.00
Rural			0.73^**^ [0.60, 0.88]	0.77^**^ [0.64, 0.93]
**Geographical sub‐regions**				
Southern Africa			1.00	1.00
Central Africa			0.31^***^ [0.16, 0.59]	0.39^**^ [0.20,0.77]
Eastern Africa			0.36^***^ [0.20, 0.66]	0.36^**^ [0.19, 0.69]
Western Africa			0.33^***^ [0.18, 0.60]	0.42^**^ [0.22, 0.79]
**Random effect model**				
PSU variance (95% CI)	5.986 [4.952, 7.236]	5.603 [4.627, 6.785]	5.818 [4.803, 7.047]	5.606 [4.624, 6.795]
ICC	0.645	0.630	0.639	0.630
Wald chi‐square	Reference	199.65 (<0.001)	164.97 (<0.001)	284.19 (<0.001)
**Model fitness**				
Log‐likelihood	−198,779.73	−191,721.94	−192,973.4	−189,110.44
AIC	397,563.5	383,479.9	385,966.8	378,272.9
N	12,787	12,787	12,787	12,787
Number of clusters	1014	1014	1014	1014

*Note:* 1.00 = reference category.

Abbreviations: AIC, Akaike information criterion; aOR, adjusted odds ratios; CI, confidence interval; ICC, intra‐class correlation; PSU, primary sampling unit.

* *p* < 0.05.

** *p* < 0.01.

*** *p* < 0.001.

AGYW who listened to the radio were more likely than those who did not to seek healthcare for STIs [aOR = 1.20; 95% CI = 1.05, 1.38]. AGYW who did not encounter a problem with getting permission to go to the health facility had higher odds of seeking healthcare for STIs [aOR = 1.25; 95% CI = 1.02, 1.54] than their counterparts who had a problem. Similarly, the odds of seeking treatment for STIs were higher among AGYW who did not have a problem with not wanting to go alone [aOR = 1.25; 95% CI = 1.03, 1.50] compared to those who encountered a problem. However, the likelihood of seeking healthcare for STIs was significantly lower among AGYW living in rural areas [aOR = 0.77, 95% CI: 0.64–0.93] compared to urban areas. There were some geographic disparities in the HSB of women with STIs. Compared to AGYW in Southern Africa, the odds of health seeking for STIs were lower in Central [aOR = 0.39; 95% CI = 0.20, 0.77], Eastern [aOR = 0.36; 95% CI = 0.19, 0.69], and Western Africa [aOR = 0.42; 95% CI = 0.22, 0.79].

## 4 Discussion

This study examined the HSB of AGYW with STIs in SSA. We found that only 54.31% of AGYW sought healthcare for STIs, which aligns with findings from East Africa [17] and Iran [24]. Our finding may be a reflection of differences in accessibility to healthcare facilities and services, socioeconomic disparities, and the knowledge level of women in SSA on STIs [16]. It is also possible that women may not consider STI symptoms as requiring serious attention until they advance. Nevertheless, the multilevel regression analysis performed in this study provides insights into the factors that significantly determined the HSB of AGYW with STIs in SSA.

Age emerged as one of the most significant factors associated with HSB for STIs. Compared to adolescents, young women reported a higher likelihood of seeking advice/treatment for their STIs. This is supported by previous studies conducted in Nigeria [25] and East Africa [17] that found the odds of seeking advice/treatment for STIs to be higher among young women than among adolescents. Unlike adolescents who often tend to be naïve and lack experience in relation to sexual and reproductive health matters [26], young women are more aware and informed about STIs, their symptoms, and where to find treatment. Another perspective on this result could be that, although adolescents may have adequate information about STIs and their treatment, they may feel shy, embarrassed, or afraid to seek advice and treatment for STIs [27]. However, young women may be more assertive in seeking advice and treatment. This may be grounded in the traditional and cultural norms that present sex and its related outcomes (e.g., STIs) as a taboo for adolescents.

Having a formal education was a factor that was associated with a higher probability for AGYW to seek advice/treatment for STIs. This result is consistent with earlier findings [16, 17, 28] that found STI‐related HSB to be higher among women with formal education than among those without formal education. Education has a wider scope that extends beyond equipping individuals with the necessary knowledge and skills to make informed decisions concerning their health [29]. It is also likely to affect an individual's socioeconomic status, which in turn influences their access to healthcare services. This makes it more likely for AGYW with higher educational attainment to be employed and afford the related costs associated with seeking advice/treatment for STIs. It is also possible that the result may be a demonstration of how education empowers women to make autonomous healthcare decisions [16]. Moreover, having higher educational attainment often translates to a greater tendency to be exposed to information regarding the need for appropriate HSB and the dangers associated with not seeking care [18].

AGYW who belonged to the richest wealth index and those who were currently employed during the survey exhibit a higher tendency to seek advice or treatment for STIs. Our finding corroborates studies conducted in Ghana [2] and SSA [18]. Our findings could be that AGYW from wealthy households and those in employment may have greater access to healthcare services, including the ability to pay for healthcare and transportation to healthcare facilities [18]. Moreover, women from high wealth status households may also have more health literacy and knowledge about the benefits of seeking treatment for STIs and, they might be more equipped to make decisions regarding their health [30]. Conversely, women in the poorest wealth index and those who were unemployed may encounter financial difficulties while attempting to get healthcare treatments and, therefore, prioritise other necessities above STI‐related healthcare.

The odds of seeking treatment was higher among AGYW who were covered by health insurance. Health insurance can lessen financial barriers to receiving healthcare services [31], such as the expense of consultations, laboratory testing, and drugs. This may encourage women to seek treatment for STIs, particularly if they are having symptoms or have been notified of their STI status. Possessing health insurance may also be indicative of a higher socioeconomic position, which is associated with greater access to healthcare facilities and increased awareness of the importance of obtaining treatment for STIs [18].

We found that problems with accessibility were not significant in predicting the HSB of AGYW. This was reflected in our findings that problems with getting money needed for treatment and distance to the health facility were not significantly associated with the STI HSB. This is inconsistent when compared to previous studies [17, 18] that have found these two factors to be significant. In certain cultures, women are obliged to ask permission from their husbands or other male family members before seeking healthcare [32, 33], thus resulting in delayed or limited access to STI care, leading to complications and worse health consequences. Therefore, the observed association could be a reflection of the autonomy of women to seek healthcare for STIs.

AGYW's exposure to listening to the radio was associated with an increased likelihood of seeking treatment for STIs. This finding is in line with a previous study conducted in Ghana [13], which reported that frequent listening to the radio was associated with a higher odds of seeking treatment for STIs. The observed association is not surprising, as there is a preponderance of literature that supports the argument that radio is an effective tool or medium for awareness campaigns and health education about STIs and their treatments [13, 34]. This implies that listening to the radio may have provided the women with the necessary information to make an informed decision to seek healthcare for their STIs.

Living in less privileged areas was significantly associated with lower odds of seeking advice/treatment for STIs. Seidu et al.’s [18] study supports this finding. We argue that this finding can be interpreted from several perspectives. First, healthcare facilities and services are scarcer in rural locations than in urban areas [17, 35]. Moreover, transportation in rural locations is sometimes limited and costly [36], making it difficult for individuals to commute to healthcare facilities, especially for non‐emergency treatments. Additionally, societal and cultural norms prevalent in rural areas may stigmatise seeking treatment for STIs, causing individuals to hesitate to seek care. In addition, economic discrepancies between urban and rural locations can influence HSB, as rural residents may have lower incomes and less access to health insurance [37], rendering healthcare services expensive.

Finally, our study revealed significant differences in the likelihood of seeking healthcare for STIs across the dimension of geographic location. This was evident in the significantly low odds reported in Central, Eastern, and Western Africa compared to AGYW in Southern Africa. The result aligns with a related study from SSA [18]. Sub‐regional studies are recommended to ascertain the specific factors associated with the variations in HSB for STIs among AGYW. However, Seidu et al. [18] have postulated that the prevalence of political and civil upheaval, as well as terrorism, may account for the low odds observed among AGYW in Central and Eastern Africa. Such unrests result in instabilities in the healthcare system and may influence HSB for STIs.

This study was limited in a number of ways. Due to the cross‐sectional nature of the DHS, no causality can be established. Moreover, as a secondary analysis, we were limited to only the variables that were available in the dataset. This means that key confounders, such as the quality of health services, affordability of STI health services, and attitudes of healthcare providers, could not be included in statistical modelling. Given that the outcome variable was self‐reported, there is a tendency for recall bias and social desirability bias.

### 4.1 Implications for Policy and Practice

The findings from this study underscore a need for more efforts to improve STI healthcare seeking in rural areas and among AGYW of low wealth status, as well as among those with no formal education. Practically, this can be achieved by intensifying awareness campaigns and health education programmes about the relevance of seeking healthcare for STIs. To increase healthcare access for AGYW, policymakers should examine policies to extend health insurance coverage and give financial support to individuals who are not insured. Given the significance of access to radio in this study, governments across the sub‐Saharan African countries must work through their appropriate agencies and departments to leverage radio as a medium for campaigns that support HSB for STIs.

## 5 Conclusion

Our study has shown that about half of AGYW with STIs did not seek treatment for the STIs. However, variations exist in healthcare seeking for STIs across the countries included in the study. The factors that supported a higher probability of seeking healthcare were being aged 20‐24, cohabiting, belonging to a high wealth index, having a formal education, listening to the radio, having no problem with getting permission, and having health insurance coverage. Residing in rural areas and in either Central, Eastern or Western Africa was associated with a lower likelihood of seeking healthcare for STIs. There is also a need to invest in media campaigns to raise awareness and encourage behaviour that is consistent with seeking healthcare.

## Author Contributions

**Bright Opoku Ahinkorah**: conceptualization, methodology, supervision, investigation, writing – original draft, writing – review and editing. **Richard Gyan Aboagye**: investigation, formal analysis, writing – original draft. **Irene Esi Donkoh**: writing – original draft. **Joshua Okyere**: investigation, formal analysis, writing – original draft. **Sanni Yaya**: conceptualization, investigation, writing – original draft, methodology, supervision, writing – review and editing.

## Conflicts of Interest

The authors declare no conflicts of interest.

## Transparency Statement

Sanni Yaya affirms that the manuscript is an honest, accurate and transparent account of the study being reported; that no important aspects of the study have been omitted, and that any discrepancies from the study as planned have been explained

## Data Availability

All analysed data are freely available to the public through https://dhsprogram.com/data/available‐datasets.cfm.
